# Analysis of laboratory data transmission between two healthcare institutions using a widely used point-to-point health information exchange platform: a case report

**DOI:** 10.1093/jamiaopen/ooae032

**Published:** 2024-04-24

**Authors:** Hung S Luu, Walter S Campbell, Raja A Cholan, Mary E Edgerton, Andrea Englund, Alana Keller, Elizabeth D Korte, Sandra H Mitchell, Greg T Watkins, Lindsay Westervelt, Daniel Wyman, Stephen Powell

**Affiliations:** Department of Pathology, University of Texas Southwestern Medical Center, Dallas, TX 75390, United States; Department of Pathology and Microbiology, University of Nebraska Medical Center, Omaha, NE 68198, United States; Deloitte Consulting LLP, Washington, DC 20004, United States; Department of Pathology and Microbiology, University of Nebraska Medical Center, Omaha, NE 68198, United States; Department of Pathology and Microbiology, University of Nebraska Medical Center, Omaha, NE 68198, United States; Synensys, LLC, Peachtree, GA 30269, United States; Deloitte Consulting LLP, Washington, DC 20004, United States; JP Systems, Inc., Clifton, VA 20124, United States; Deloitte Consulting LLP, Washington, DC 20004, United States; JP Systems, Inc., Clifton, VA 20124, United States; Synensys, LLC, Peachtree, GA 30269, United States; Synensys, LLC, Peachtree, GA 30269, United States

**Keywords:** health information exchange, health information interoperability, clinical laboratory information systems, patient safety, data quality

## Abstract

**Objective:**

The objective was to identify information loss that could affect clinical care in laboratory data transmission between 2 health care institutions via a Health Information Exchange platform.

**Materials and Methods:**

Data transmission results of 9 laboratory tests, including LOINC codes, were compared in the following: between sending and receiving electronic health record (EHR) systems, the individual Health Level Seven International (HL7) Version 2 messages across the instrument, laboratory information system, and sending EHR.

**Results:**

Loss of information for similar tests indicated the following potential patient safety issues: (1) consistently missing specimen source; (2) lack of reporting of analytical technique or instrument platform; (3) inconsistent units and reference ranges; (4) discordant LOINC code use; and (5) increased complexity with multiple HL7 versions.

**Discussion and Conclusions:**

Using an HIE with standard messaging, SHIELD (Systemic Harmonization and Interoperability Enhancement for Laboratory Data) recommendations, and enhanced EHR functionality to support necessary data elements would yield consistent test identification and result value transmission.

## Introduction

Despite emphasis on health information exchange (HIE), interoperability of pathology data has not been sufficiently characterized. This case report evaluates the fidelity of laboratory data when shared between 2 healthcare institutions via a commonly used HIE construct to provide electronic access to laboratory data generated across both facilities.

Lack of access to relevant clinical data sourced from multiple providers can result in potential patient harm.^1^ Successful laboratory data interoperability requires accuracy in data exchange along with recognition of comparable test types and results. HIE platforms were created to manage accurate exchange of multiple data values comprising results and associated metadata.^2^^,^^3^ Standard ontologies have been developed to ensure test comparability. The primary goal of the Systemic Harmonization and Interoperability Enhancement for Laboratory Data (SHIELD), a collaborative community, has been to achieve a level of laboratory data interoperability that enhances patient care and prevent potential patient safety issues. Semantic standards were evaluated by SHIELD public workshops in 2015 and 2016 with the consensus that results of in vitro diagnostic (IVD) tests were best represented by a combination of terms. Laboratory tests and results can be thought of as a question/answer pair where the questions asked can be represented by LOINC terms,^4^ while the answer for qualitative tests can be described with SNOMED CT codes (Systematized Nomenclature of Medicine—Clinical Terms).^5^ Quantitative values would ideally be characterized by their units of measure using UCUM (Unified Codes for Units of Measure), although it is recognized that further development is required to address current limitations of UCUM. Test method and the specific device would be described using the Device Identifier component of the Unique Device Identification (UDI) system.^6^ While the UDI system would address most FDA-cleared IVD tests, laboratory developed tests (LDTs) or tests designed, manufactured, and used within a single laboratory as well as assays approved with an Emergency Use Authorization lack UDIs and will require and alternative strategy. It has been postulated that, when combined with LOINC, the UDI could provide the specificity needed to prevent aggregation and trending of non-harmonized laboratory results.^7^ Combining standardized nomenclature and accurate data transmission would achieve interoperability. We demonstrate inadequacies in the current state of interoperability by exploring information exchange within and between 2 non-affiliated healthcare institutions for a specified set of laboratory test values for a single (test) patient.

## Materials and methods

### Setting

**Figure 1.**
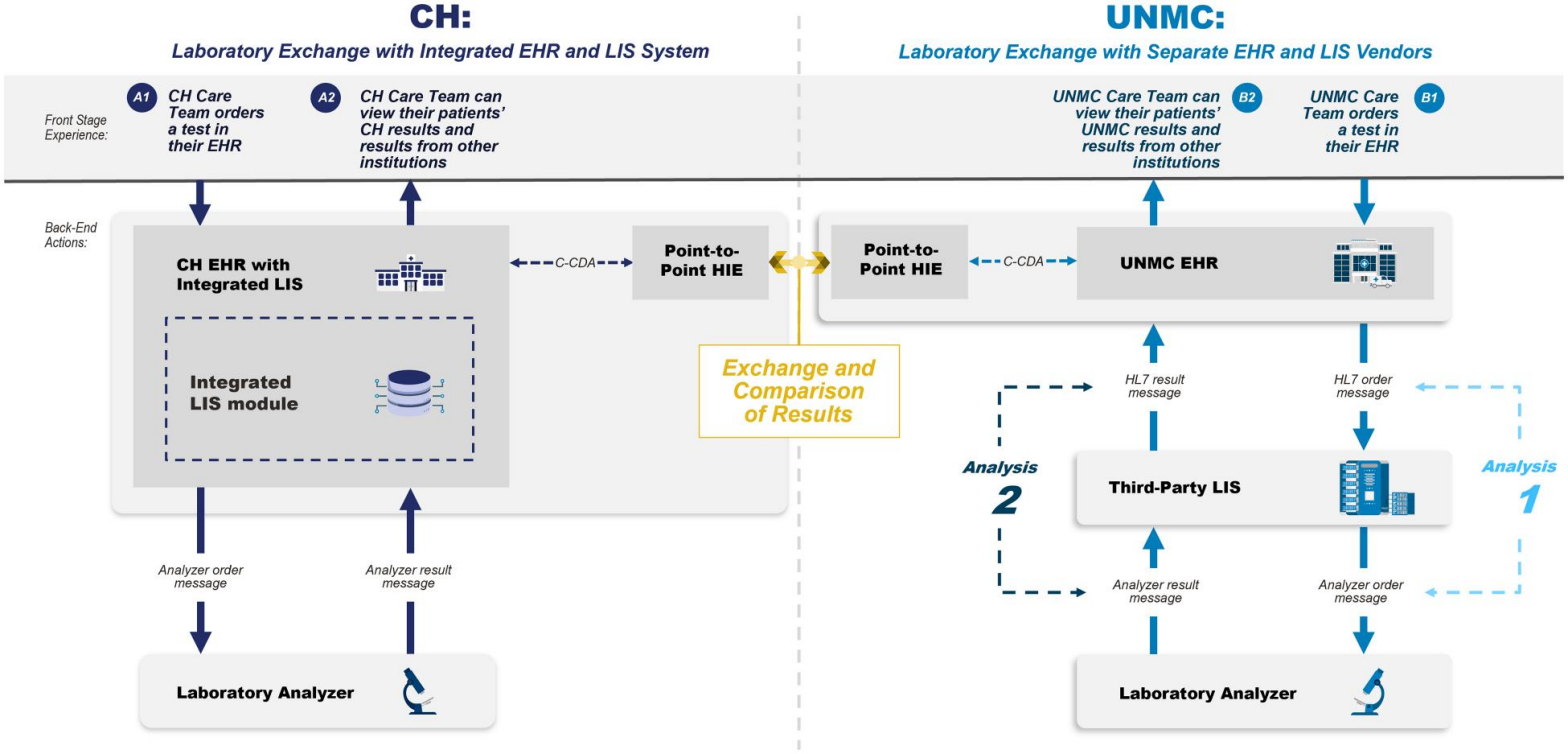
Laboratory data exchange at 2 institutions. Nine laboratory tests were selected to be resulted for a simulated patient and are representative examples of high priority clinical laboratory tests. The test results were transferred to the other institution through a widely used point to point health information exchange platform. We compared the gaps and similarities in the laboratory test information displayed in the sending EHR system with the information displayed in the receiving EHR. Health Level Seven International (HL7) Version 2 messages between the EHR and LIS (Analysis 1) and analyzer messages between the LIS and IVD analyzers (Analysis 2) were obtained from UNMC for each of the 9 test orders and test results and examined for structure and content. Further details of the Analysis 1 and Analysis 2 of the HL7 messages are available in the supplemental materials.

Two major laboratories, University of Nebraska Medical Center (UNMC) in Omaha, Nebraska, and Children’s Health (CH) in Dallas, TX, participated in this case report. UNMC is a 735-bed tertiary care facility with 11 million laboratory tests performed annually. CH is a 562-bed pediatric hospital whose laboratory performs 1.6 million tests annually. Both laboratories are heavily used as reference testing centers by as many as 300 other health facilities. Both institutions use Epic (Verona, WI) as their electronic health record (EHR) to display results to the healthcare provider. The laboratory information system (LIS) for CH is Epic Beaker. The LIS for UNMC is CliniSys (Tucson, AZ). Figure 1 illustrates the flow of laboratory data across individual analyzers, the LIS, the EHR, and then via the HIE to a separate EHR.

### Laboratory data transmission through HIE

A simulated patient with the same identifiers (ie, name, gender, date of birth, and address) was created in both EHRs. Nine clinically actionable laboratory tests were selected to be resulted for the simulated patient at each institution and include quantitative [D-Dimer, High-Sensitivity Troponin I, Ammonia, Complete Blood Count (CBC), Complete Metabolic Panel (CMP), Hemoglobin A1C (Hb A1c), Prothrombin Time (PT) and International Normalized Ratio (INR), Activated Partial Thromboplastin Time (aPTT)] and qualitative (Urine Drug Screen) tests.

The sample values, obtained from the College of American Pathologists (CAP) proficiency testing (PT) peer group data, are the mean values for the analytes reported by laboratories using the same instrumentation from testing performed on aliquots of a single reference specimen.^8–11^ Different IVD instruments were used at each of the 2 locations for all tests except for the CBC.

The results were transferred from the sending to the receiving institution via a widely used point-to-point HIE platform.^12^ Once transmitted to the receiving institution, they appeared in 2 places in the receiving EHR: (1) the Encounter Summary (ie, summary of a clinic visit) as viewed from the sending institution and (2) the Lab Tab view of the receiving EHR. The Encounter Summary is a narrative that typically includes progress notes along with test results. In the Lab Tab, external results appear as individual line entries. These displays at each institution were examined to document their completeness with respect to the following information for each test generated both from their own laboratory and as reported from the sending laboratory: patient ID, report date, test performed, specimen source, test result, units of measure, reference range, abnormal flag, specimen exception, annotation, IVD instrument and method, and performing laboratory.

### Internal laboratory order and result messaging interface

Messaging between the EHR, LIS, and the performing analyzer was available for 1 of the 2 sites and was also examined to evaluate the consistency of the transmitted information for each of the 9 tests. Health Level Seven International (HL7) Version 2 (V2) messages were generated between the EHR and LIS for each of the 9 test orders and results. Message transactions between the LIS and performing IVD instruments used a local, non-standard dictionary.

## Results

### Test value comparison

Table 1 compares the tests resulted in each system including the LOINC code in the LIS, sample value, units of measure, reference range, and instrumentation. Except for the hematology analyzers, all of the instrumentation for the other assays differed between the laboratories. The CMP values were comparable between the 2 institutions because the IVD manufacturers had harmonized their results by calibrating their instruments and reagents to a standardized material. The remaining 6 quantitative assays were selected for inclusion in Table 1 due to the fact that they had not been harmonized by the IVD manufacturers and produced strikingly variable results despite appropriately sharing the same LOINC codes. Discordance was identified between 3 of the 6 LOINC codes examined for each institution. The reasons for the discordance included use of deprecated codes, selecting the incorrect system axis and incorrect method. The sample value reported for each test is obtained from proficiency testing peer group data and is the average mean of numerical results for the groups of laboratories utilizing the same instrumentation and derived from aliquots of the same specimen.^8–11^

**Table 1.** Test result comparison between UNMC and CH.

Test/institution	LOINC code	Sample value	Units of measure	Reference range	Instrumentation	Reason for mismatch of LOINC codes
D-Dimer						
UNMC	48065-7; Fibrin D-dimer FEU [Mass/volume] in platelet poor plasma	278.721	ng/mL FEU	<500	ACL TOP 500 CTS	
CH	48065-7; Fibrin D-dimer FEU [Mass/volume] in platelet poor plasma	0.368	mcg/mL FEU	0.00-0.50	Stago STA-R Max Coagulation Analyzer	
High-sensitivity troponin I						
UNMC	89579-7; Troponin I.cardiac [Mass/volume] in serum or plasma by high sensitivity method	14	pg/mL	<21 pg/mL	Beckman Coulter AU5800	CH using LOINC for standard Troponin I instead of high-sensitivity
CH	10839-9; Troponin I.cardiac [Mass/volume] in serum or plasma	44.1	pg/mL	≤45.00 pg/mL	Siemens Atellica	
Ammonia						
UNMC	1839-0; Ammonia [Moles/volume] in blood	419.7	μmol/L	11-32	Beckman Coulter AU5800	
CH	1839-0; Ammonia [Moles/volume] in blood	425	μmol/L	23-46	Siemens Atellica	
Hemoglobin A1C						
UNMC	55454-3; Deprecated Hemoglobin A1c in blood	5.92	%	4.0%-6.0%	Trinity Biotech Boronate Affinity Chromatography	UNMC using deprecated Hb A1c code instead of active code
CH	4548-4; Hemoglobin A1c/Hemoglobin.total in blood	5.74	%	<5.7%	Abbott Afinion 2	
Prothrombin time and international normalized ratio						
UNMC	5964-2; Prothrombin time (PT) in blood by coagulation assay	11.61	seconds	9.9-13.7 seconds	ACL TOP 500 CTS	UNMC using LOINC for whole blood (international assay) instead of platelet-poor plasma
CH	5902-2; Prothrombin time (PT)	13.44	seconds	12-15.2 seconds	Stago STA-R Max Coagulation Analyzer	
Activated partial thromboplastin time						
UNMC	14979-9; aPTT in Platelet poor plasma by coagulation assay	28.2	seconds	22.0-36.0 seconds	ACL TOP 500 CTS	
CH	14979-9; aPTT in Platelet poor plasma by coagulation assay	29.8	seconds	21.3-38.8 seconds	Stago STA-R Max Coagulation Analyzer	

*Abbreviations:* CH, Children’s Health; FEU, Fibrinogen Equivalent Units; UNMC, University of Nebraska Medical Center.

### Laboratory data display and transmission through HIE

Table 2 compares the completeness of the displayed data at each of the institutions for (1) the locally sourced results; (2) the sending institution results displayed in the Encounter Summary; and (3) the sending institution results displayed in the Lab Tab view. All 9 tests were evaluated. Locally sourced results displayed in the LIS and EHR of the sending institution were completely reported except for instrument and method. Instrument and method were inconsistently displayed in the sending LIS depending on the LIS vendor and not displayed at all in either the sending or receiving EHR. Note that a patient-facing healthcare provider making treatment decisions does not have access to the LIS.

**Table 2.** Comparison of available data elements at UNMC and CH.

Institution/view	Patient ID^a^	Report date^a^	Test performed^a^	Specimen source^a^	Test result^a^	Units of measure^a^	Reference range^a^	Abnormal flag^a^	Result comment^a^	Specimen exception notation^a^	Method	Laboratory^a^
Laboratory result data element summary for UNMC												
** **UNMC EHR view of UNMC results	Present	Present	Present	Present	Present	Present	Present	Present	Present	Present	**Not present**	Present
** **CH EHR encounter summary of UNMC results	Present	Present	Present	Present	Present	Present	Present	Present	Present	Present	**Field available**	Present
** **CH EHR—Lab Tab of UNMC results	Present	Present	Present	**Not present**	Present	Present	Present	Present	Present	Present	**Not present**	Present
** **UNMC LIS view of UNMC results	Present	Present	Present	**Not present**	Present	Present	Present	Present	Present	Present	Present	Present
Laboratory result data element summary for CH												
** **CH EHR view of CH results	Present	Present	Present	Present	Present	Present	Present	Present	Present	Present	**Not present**	Present
** **UNMC EHR encounter summary of CH results	Present	Present	Present	Present	Present	Present	Present	Present	Present	Present	**Field available**	Present
** **UNMC EHR—Lab Tab of CH results	Present	Present	Present	**Not present**	Present	Present	Present	Present	Present	Present	**Not present**	Present
** **CH LIS view of CH results	Present	Present	Present	**Present**	Present	Present	Present	Present	Present	Present	Present	Present

a CLIA required data elements.

*Abbreviations:* CH, Children’s Health; EHR, Electronic Health Record; LIS, Laboratory Information System; UNMC, University of Nebraska Medical Center.

**Figure 2.**
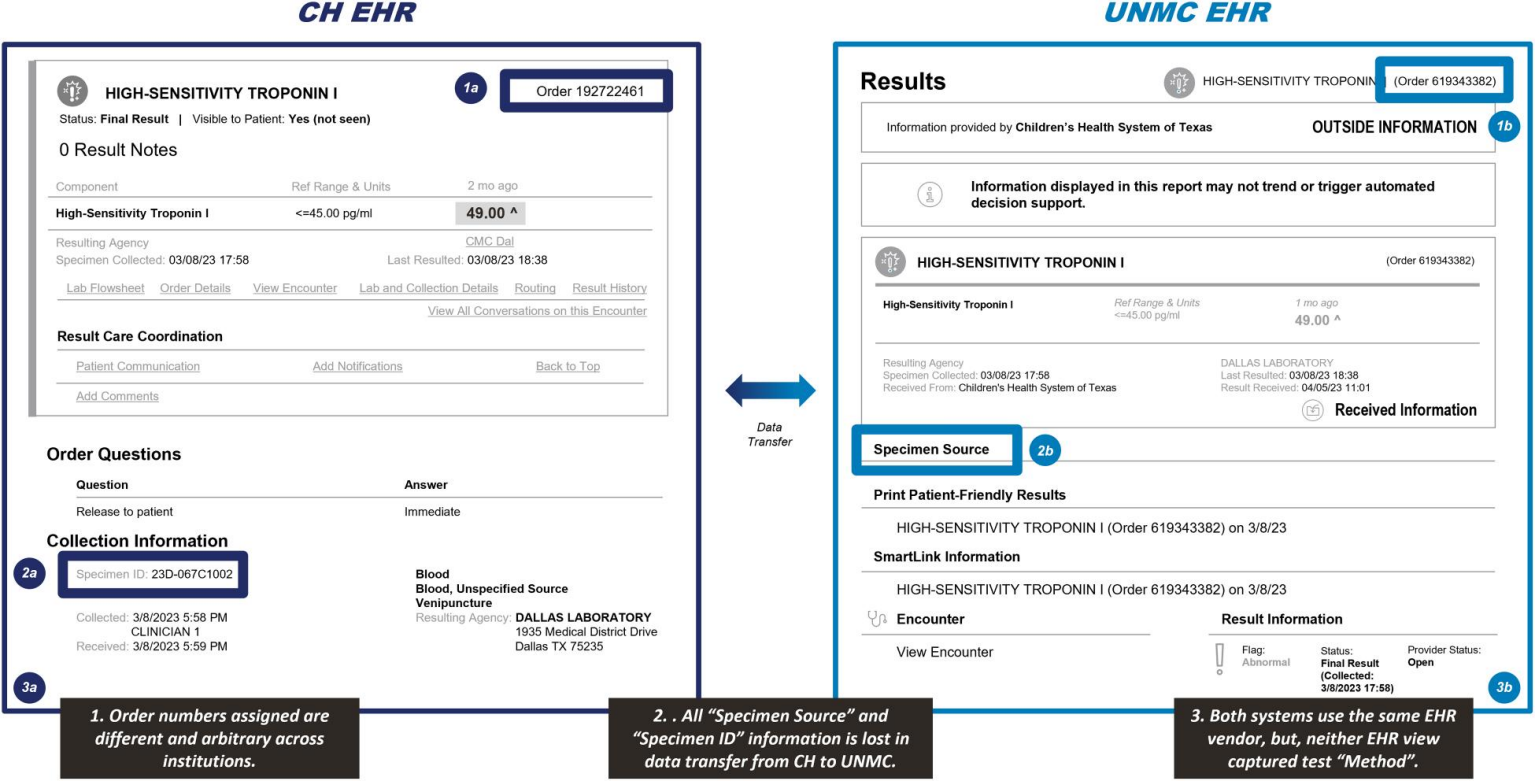
Comparison of electronic health record data at children’s health and University of Nebraska Medical Center.

Specimen source is visible in the Encounter Summary, but this information is lost in the Lab Tab view (see Figure 2). There is an empty field available to display method information in the Encounter Summary, but none in the Lab Tab view. There is an arbitrary order number created for the transferred results that does not match the order number for the result at the sending institution.

### Internal laboratory order and result messaging interface

HL7 V2 messages between the EHR and LIS and analyzer messages between the LIS and IVD instruments were generated by UNMC for each of the 9 test orders and test results. Review of these identified not only the inconsistent use of LOINC codes, but also that different HL7 V2 versions were being used at the same institution for ordering and resulting.

## Discussion

Utilizing an HIE with standard messaging and following the SHIELD recommendations (ie, consistently using SNOMED CT, LOINC, UCUM, and UDI), it has been postulated that lab data interoperability can be achieved.^12^ Lab data interoperability is defined as accurate and complete exchange of test name and test result values while realizing consistency in test values from the same specimen.

Completeness of clinical laboratory data reporting is defined by the federal regulations in the Clinical Laboratory Improvement Amendments of 1988 (CLIA). CLIA has oversight over information sent from “the laboratory (ie, its LIS) to the final report destination which is typically the first downstream interfaced system (eg, EHR).” It does not govern laboratory data exchanged through HIE platforms between healthcare institutions.^13^

CLIA regulations require that the reported results must include the following data: unique identifiers for the patient, name and address of the laboratory location where the test was performed, test performed along with the test report date, specimen source when appropriate, test results with associated units of measurement or qualitative interpretation, and any information regarding the condition and disposition of specimens that does not meet the laboratory’s criteria for acceptability.^14^

Analysis of the display of the transferred results showed that specimen source was consistently missing in the receiving EHR Lab Tab display although available in the Encounter Summary narrative. Considering that different clinical specimens can yield different results, for example, different rates of detection for SARS-CoV-2 during the COVID-19 pandemic,^15^ this absence of information in the Lab Tab view can result in potential patient safety issues. Another example would be Herpes Simplex Virus (HSV) polymerase chain reaction (PCR) performed on multiple skin lesions where it would be very important for the clinician to know which sites were positive. HSV PCR can also be performed on cerebral spinal fluid or the eye; the clinical implications for positive results in these sites are very different than a skin lesion and therefore need to be clearly communicated in the test result.

Differences in either analytical technique, instrument, or platform were not reported even though the name of the test at each institution was the same. The outcome was significant variability in results for a single named test (see Table 1). In the case of high-sensitive troponin I, a user interpreting the result of an externally generated value based on their internal reference range could result in the misdiagnosis of the patient as experiencing a cardiac event, leading to unnecessary further evaluation and treatment, or failure to diagnose an acute myocardial infarction due the magnitude of difference between the 2 values. There were also multiple instances of differences between the institutions in the units of measure used to report a test result. This could also lead to errors in medical treatment if not brought to the attention of the healthcare provider scanning the record.

There was significant discordance in LOINC codes for the same test at both institutions. Reasons for this included use of deprecated codes, the incorrect system axis, or incorrect method. Errors in applications of LOINC codes have been reported in prior studies.^16–18^ Tests that were appropriately assigned to the same LOINC code could produce very different quantitative values due to the different instrumentation and method used to derive the results as evident in Table 1. Based on current usage patterns, the hypothesis of being able to disaggregate different tests based on LOINC codes is invalidated.

In addition to these results that impact comparability and the ability to interpret the lab data, data transmission is not sufficiently complete or accurate to meet CLIA requirements. This occurs not only in exchange between EHRs, but, as demonstrated in the Results section, it is exacerbated using multiple versions of HL7 within a single integration pattern. The differences between HL7 V2.3.1 and V2.5.1 are that deprecated fields are not supported and some value sets are changed or replaced. Optionality, the number of data elements within the standard that are designated as “optional” and might either be populated or left NULL in a conformant message instance, varies between HL7 versions. The practical implication of a message standard’s optionality is that it increases the number of data elements that interoperating partners must recognize and implement identically. Greater optionality can increase the time and effort required to build a functioning interface to eliminate risk to the patient.^19^

Exchange standards such as the SHIELD recommendations are only part of the solution. EHR and LIS functionality must be standardized to allow for consistent capture, transmission, and display of essential data elements. In current practice, most EHR and LIS systems are not equipped to capture and display instrument/method information, performing laboratory location, specimen source (other than microbiology tests) or information regarding the condition and disposition of specimens that does not meet the laboratory’s criteria for acceptability as discrete data fields. This information, if provided at all, is often included as free text in the result comment. Lack of a discrete field means that inclusion of these data elements is inconsistent with a variable format. It makes finding the information when it is included challenging as it does not appear in a consistent location or in a standardized format.

Until these problems are addressed, simple HIE platforms using locally selected codes of test names and results, without regard to changes in units of measure, and without consideration of variability across instruments, can lead to potential patient safety issues when aggregating data across multiple laboratory sources. Additional functionality also exists to fully map external results in the EHR and allow results from other institutions to trend on the same line as locally produced test results in Result Review. In Result Review, test results appear in a spreadsheet-like format and certain information such as units of measure and reference ranges are not displayed by default. Clinicians are able to see the suppressed information by hovering over the result value and may not be able to readily distinguish local laboratory results from results imported from other institutions. Laboratory testing occupies a prominent place in healthcare.^20^ While interoperability of laboratory data can improve patient care,^3^ if it is not sufficiently complete, accurate, and comparable, then attempts at data exchange across institutions can result in potential patient safety issues.^21^

Despite this, there are few government mandates that address the lack of standards in reporting of laboratory data.^16^^,^^17^ While health care stakeholders, including patients, might assume that they can receive comparable results from different laboratories, this is not the case.^22^ Different analytical methods alone introduce significant variability. Thus, it should never be assumed that the differently sourced test values can be directly compared. In addition to requirements for the display of laboratory data in the EHR to be accurate, complete, easily accessible, and readily interpreted, the display must also be able to make the healthcare provider aware of the implications of test results sourced from different laboratories.

### Limitations

While the 9 tests selected are representative examples of high priority clinical laboratory tests conducted on various IVD instruments and include differing test results, units of measure, reference ranges, and methods, they also are some of the lowest complexity to perform and do not require pathologist interpretation. They are also all FDA-cleared assays with an assigned UDI. The results reported fell within the normal range for the assays, and we did not study results that fall either at the edge or outside the reportable range of the performing instruments. The 2 institutions were also selected because they both utilize the same EHR system that developed the HIE platform. The exchange of laboratory data across 2 institutions utilizing different EHR systems across a state or other HIE may yield even more loss of data and meaning as the HIE may not have been uniquely designed to consume and transmit information from a specific EHR as is the case in our case study. The complexity and optionality challenges would only be expected to magnify with the exchange of data between heterogeneous systems and the interoperability gaps documented in this study could only be expected to worsen with more complicated tests and results.

## Disclaimer

This article reflects the views of the authors and should not be construed to represent views or policies of the US Food and Drug Administration or the participating institutions.

## Supplementary Material

ooae032_Supplementary_Data

## Data Availability

The data underlying this article will be shared on reasonable request to the corresponding author.
