# Supplementary figures and images for: Analysis of laboratory data transmission between two healthcare institutions using a widely used point-to-point health information exchange platform: a case report
Source: JAMIA Open. 2024 Apr 24;7(2):ooae032. doi: 10.1093/jamiaopen/ooae032 (PMC11042873; doi:10.1093/jamiaopen/ooae032)

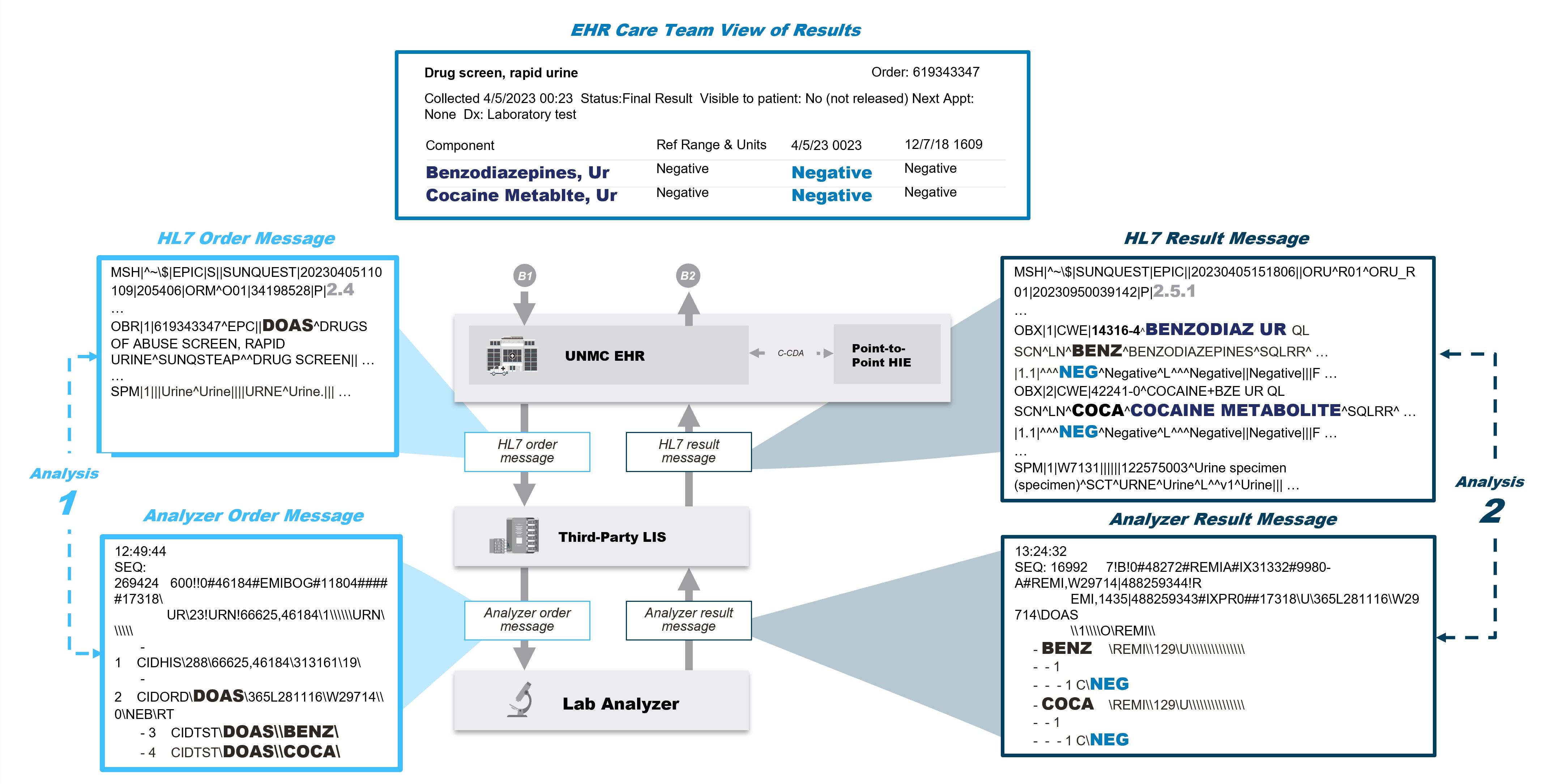

Supplement: ooae032_Supplementary_Data [file ooae032_supplementary_data.jpeg]
